# [Retracted] TFAP4 promotes the growth of prostate cancer cells by upregulating FOXK1

**DOI:** 10.3892/etm.2024.12754

**Published:** 2024-10-29

**Authors:** Yuan Gu, Jiujin Jiang, Chaozhao Liang

Exp Ther Med 22:1299, 2021; DOI: 10.3892/etm.2021.10734

Following the publication of this paper, it was drawn to the Editor’s attention by a concerned reader that the ‘shNC’ data panel for the Transwell invasion assay data shown in Fig. 3B (for the DU145 cell line) on p. 5 were strikingly similar to data that had already been published in different form in another article written by different authors at a different research institute [Zuo K, Zhao Y, Zheng Y, Chen D, Liu X, Du S and Liu Q: Long non-coding RNA XIST promotes malignant behavior of epithelial ovarian cancer. Onco Targets Ther 12: 7261-7267, 2019].

Owing to the fact that the contentious data in the above article had already been published prior to its submission to *Experimental and Therapeutic Medicine,* the Editor has decided that this paper should be retracted from the Journal. The authors were asked for an explanation to account for these concerns, but the Editorial Office did not receive a reply. The Editor apologizes to the readership for any inconvenience caused.

